# Rodent Abundance Dynamics and Leptospirosis Carriage in an Area of Hyper-Endemicity in New Caledonia

**DOI:** 10.1371/journal.pntd.0001361

**Published:** 2011-10-25

**Authors:** Julie Perez, Fabrice Brescia, Jérôme Becam, Carine Mauron, Cyrille Goarant

**Affiliations:** 1 Institut Pasteur de Nouvelle-Calédonie, Réseau International des Instituts Pasteur, Laboratoire de Recherche en Bactériologie, Nouméa, New Caledonia; 2 Institut Agronomique néo-Calédonien, Diversités Biologique et Fonctionnelle des Ecosystèmes, Port Laguerre, Paita, New Caledonia; University of California San Diego School of Medicine, United States of America

## Abstract

**Background:**

Widespread but particularly incident in the tropics, leptospirosis is transmitted to humans directly or indirectly by virtually any Mammal species. However, rodents are recognized as the most important reservoir. In endemic regions, seasonal outbreaks are observed during hot rainy periods. In such regions, hot spots can be evidenced, where leptospirosis is “hyper-endemic”, its incidence reaching 500 annual cases per 100,000. A better knowledge of how rodent populations and their *Leptospira* prevalence respond to seasonal and meteorological fluctuations might help implement relevant control measures.

**Methodology/Principal Findings:**

In two tribes in New Caledonia with hyper-endemic leptospirosis, rodent abundance and *Leptospira* prevalence was studied twice a year, in hot and cool seasons for two consecutive years. Highly contrasted meteorological situations, particularly rainfall intensities, were noted between the two hot seasons studied. Our results show that during a hot and rainy period, both the rodent populations and their *Leptospira* carriage were higher. This pattern was more salient in commensal rodents than in the sylvatic rats.

**Conclusions/Significance:**

The dynamics of rodents and their *Leptospira* carriage changed during the survey, probably under the influence of meteorology. Rodents were both more numerous and more frequently carrying (therefore disseminating) leptospires during a hot rainy period, also corresponding to a flooding period with higher risks of human exposure to waters and watered soils. The outbreaks of leptospirosis in hyper-endemic areas could arise from meteorological conditions leading to both an increased risk of exposure of humans and an increased volume of the rodent reservoir. Rodent control measures would therefore be most effective during cool and dry seasons, when rodent populations and leptospirosis incidence are low.

## Author Summary

In this study, we surveyed rodents and their *Leptospira* carriage in an area where human leptospirosis is hyper-endemic. We evidenced the well-known associations between specific rodent species and particular leptospires in both mice and rats. Overall, the observed *Leptospira* prevalence was in the range 18–47% depending on species, similar to other descriptions. However, significant variations were observed both in the abundance of rodents and their *Leptospira* carriage, one hot period with heavy rain being associated with both a highest abundance and an increased prevalence. Similar meteorological conditions could lead to increased leptospires dispersal by the rodent reservoir and increased exposure of humans to risk situations (e.g. flood, mud). Because rodent control measures were demonstrated elsewhere to be cost-effective if correctly planned and implemented, this contribution to a better knowledge of rodent and leptospires dynamics provides useful information and may in turn allow to develop relevant rodent control actions aimed at reducing the burden of human leptospirosis.

## Introduction

Leptospirosis is an endemic bacterial disease in many tropical and sub-tropical areas. Various *Leptospira* strains, maintained in different animal species, are excreted in the urine of asymptomatic chronically infected individuals [1]–[3]. Humans get infected when abraded skin or mucous membranes come into contact with contaminated kidneys, urine or urine-contaminated environments [2]. The detailed epidemiology of leptospirosis, both a zoonosis and an environmental disease, both an occupational and a recreational disease, is highly complex. Though veterinary leptospirosis is often studied, little is usually known on how wild or feral Mammals contribute to leptospirosis dynamics [4]. Virtually any Mammal species can act as a reservoir of a co-adapted *Leptospira* strain [1], but among animal reservoirs, rodents are recognized as the most significant Mammal species maintaining and disseminating leptospires worldwide [2], [3], [5]. The Norway (or brown) rat *Rattus norvegicus* is notably known to be a reservoir of *Leptospira interrogans* of the serogroup Icterohaemorrhagiae, whereas the domestic mouse (*Mus musculus*) is a reservoir for *Leptospira borgpetersenii* of the serogroup Ballum [2], [3]. In New Caledonia, Mammal biodiversity is low: no native terrestrial Mammal is known, except 9 bat species (both micro- and megabats) [6]. However, four rodent species are known to be present, all resulting from importation by the early human settlements: three rat species (*Rattus exulans*, *Rattus rattus*, and *R. norvegicus*) and the domestic mouse (*M. musculus*) [7]. Leptospirosis in farm animals has been well studied (see [4] for review) but feral Mammals have not been investigated. Even though several strains and serovars are involved in human cases [8]–[10], Icterohaemorrhagiae is the most frequent serogroup, pointing to the importance of rodents as a major reservoir.

In most tropical regions where leptospirosis is known to be endemic, a seasonality is observed, with highest incidence during hot rainy periods, particularly after tropical storms and floods [11], [12] or during the monsoons [13]. During seasonal or post-cyclone outbreaks, there are particular areas of New Caledonia where leptospirosis incidence can reach 500 annual cases per 100,000 habitants [14]. Patchy distributions of leptospirosis have been described in New Caledonia [14], [15] but also in Brazil [16]–[18].

How human exposure to environmental contamination, reservoir abundance (especially rodents) and their *Leptospira* prevalence each contribute to such outbreaks and “leptospirosis hot spots” remains unknown. The aim of our study was to determine rodent abundance and the dynamics of *Leptospira* prevalence in their populations, in a site previously determined as a leptospirosis hot spot.

## Methods

### Choice of the study site and survey periods

**Figure 1:**
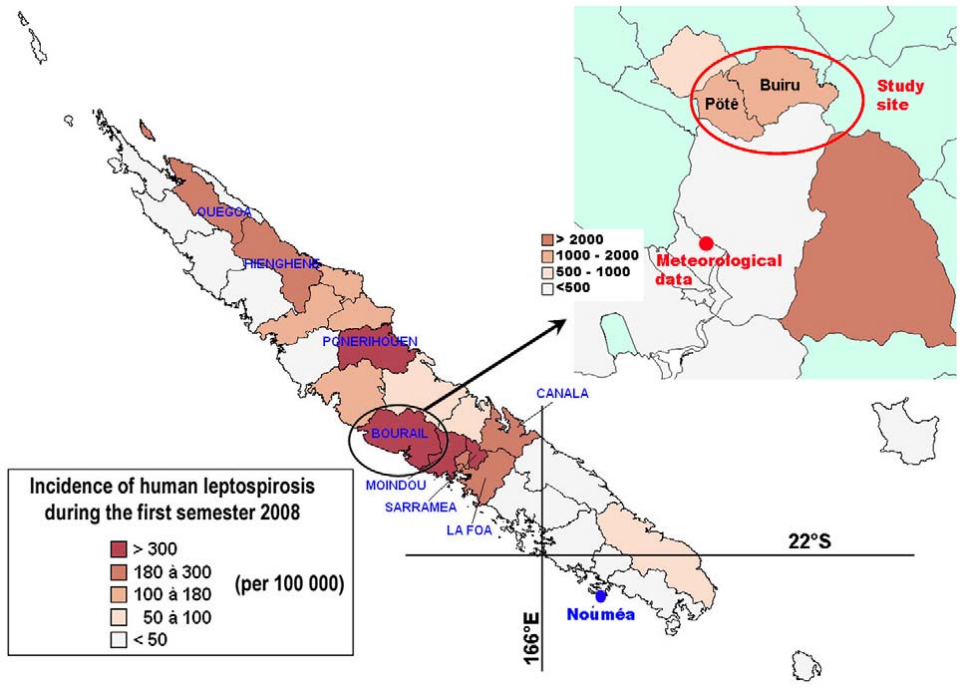
Hot spots of leptospirosis and identification of the study site and meteorological data collection point. (Map produced using PopGis, as described in the Methods section and demographic data from the Institut de la Statistique et des Études Économiques, http://www.isee.nc/.).

Based on previous studies [9], [19], the municipality of Bourail (see Figure 1) was known as a place of high incidence of leptospirosis. Using the diagnostic data of the early 2008 outbreak [14], we more precisely described the probable contamination area of these cases. Demographic data obtained from the Institut de la Statistique et des Études Économiques (http://www.isee.nc/index.html) were also used to evaluate the incidence of leptospirosis in each district. This incidence was then plotted on a map using PopGis version 1.0 (Secretariat of the Pacific Community). This allowed identifying three hot spots within the municipality of Bourail (Figure 1). The districts of Pötê and Buiru were chosen for our survey, based on their limited surface area and the good acceptance of our project by custom chiefdom and populations. These two study sites correspond to two Melanesian tribes where many outdoor activities are part of the everyday life, including fishing and bathing in freshwater streams, agriculture, maintenance of backyard pig pens, hunting (deers and wild hogs). Most of the households have one or more dogs that freely stray from the houses. Most of the inhabitants go bare foot and know the presence of rodents in and around their houses. After adequate contacts with the customs authorities, the study period was determined as a two-year period, one survey being conducted for each hot (January–April) and each cool (July–October) seasons.

### Rodent capture, identification and abundance

**Figure 2:**
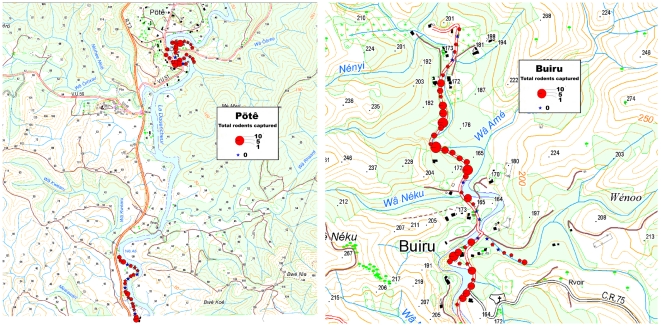
Localization of the trapping stations and cumulated trapping success over the study. Households have been colored in black. The geo-referenced map was kindly provided by DITTT, Government of New Caledonia.

Relative rodent abundance was evaluated both in the hot season (March) and during the cooler months (September-October). The sampling strategy was aimed at evaluating both the rodent abundance and the prevalence of *Leptospira* in the kidneys of the different rodent species. The method used was based on the standard index trapping technique developed in New Zealand for the study of rodents [20]: Hundred snap traps were placed by pairs (one rat- and one mouse-sized lethal snap trap) every 25 meters along a transect line close to the households within the study site. In Pötê, two transects were used with 50 traps each: one along the stream close to the households, the other one starting close to a farmer's house and extending in a cattle pasture along the same stream (Figure 2). In Buiru, the transect also extended along the streams close to the household, thus dividing at a stream confluence. Trap stations were set for 3 consecutive nights and baited with cheese and peanut butter. The processing of the trapped rats used the methods of Cunningham & Moors [20] and included the identification of species based on measurements for head-body length (HBL) and tail length (TL), assignment to a developmental stage (either adult or juvenile), and sex determination. Any animal that had been damaged by predators was identified to the species level, sex and age class was determined only if possible. Traps were checked and re-baited daily, captures and whether baits were taken or the trap sprung was also recorded. Trapping success was corrected for sprung and bait-taken traps by subtracting half a trap night for each such occurrence as described [21]. This allowed calculating an index of abundance, expressed as a number of captured animals per 100 corrected trap-nights.

### Meteorological data

Meteorological data were kindly provided by Météo-France (http://www.meteo.nc/) and corresponded to the closest automated meteorological station (Bourail). Rain (monthly accumulated rainfall) and temperature (monthly average of daily minima and monthly average of daily maxima) were plotted for the two years of the study (2009 and 2010).

### Rodent kidney samples and *Leptospira* isolation

Rodents were killed when caught by the snap traps. Each individual rodent was aseptically dissected and one kidney was immediately put in 95% alcohol for postponed DNA extraction. During the first survey (March 2008), one small piece of kidney was also aseptically transferred into a EMJH culture tube supplemented with 300 µg.ml^−1^ 5-Fluoro-Uracile as an inhibitor of contaminant bacteria [22] for *Leptospira* isolation. A few rodents captured had been attacked by cats or birds at the time of collection; some could therefore not be completely identified or dissected. These were considered for abundance calculations but not studied for *Leptospira* prevalence.

Back at the lab (at the end of the 3-day sampling period), EMJH culture tubes were incubated aerobically for 14 weeks at 30°C with weekly dark field microscopic observation. Positive cultures were immediately subcultured in fresh EMJH and then frozen at −80°C with 10% glycerol.

### DNA extraction

A small piece of each individual kidney (ca. 10 mg) was aseptically dissected, rehydrated in 3 successive ultrapure sterile water baths for 6–12 hours each. It was then grinded in 50 µL sterile Phosphate Buffer Saline, pH 7.4 (Sigma) and 180 µL ATL Buffer (QIAamp DNA mini kit, QIAGEN) using a sterile single-use Piston Pellet (Kimble Chase). DNA was then extracted using QIAamp DNA mini kit (QIAGEN) following the manufacturer's instructions for tissue. The proteinase K digestion step was set for 4 hours. Additional proteinase K (20 µL) was added to samples incompletely digested at this time and incubation prolonged until complete tissue lysis (up to 8 hours). One millilitre of each *Leptospira* culture was centrifuged and extracted using the same QIAamp DNA mini kit (QIAGEN) using the manufacturer's instructions for cultured cells. The elution volume was 100 µL for either rodent kidney or *Leptospira* isolate.

### *Leptospira* detection from rodent kidneys

*Leptospira* carriage in the rodent kidneys was assessed using two previously described diagnostic real time PCR assays, both using SYBR Green I technology, namely the detection and amplification of *lfb1* [23] or *lipL32* [24]. To check the absence of PCR inhibitors that would lead to false negative results, all kidney DNA extracts were also amplified with a “universal” 18S rDNA PCR, using primers previously described [25] and SYBR Green I technology. All oligonucleotide sequences are shown in Table S1.

### *Leptospira* identification from strains or from rodent kidneys

The *lfb1* PCR products amplified from positive kidneys were purified using the MinElute PCR Purification Kit (Qiagen) and directly sequenced as previously described [10]. For *Leptospira* isolates, a MLST scheme [26] was used as described before [10]. Alignments and phylogenies were then obtained using previously described techniques [10].

### Geographic Information System

All sampling points were referenced using a handheld GPS device (Garmin). Data were transferred to MapInfo version 7.0 (Pitney Bowes Software Inc.) on a 1/10,000 map, kindly provided by the Direction des Infrastructures, de la Topographie et des Transports Terrestres – Gouvernement de la Nouvelle-Calédonie. All captured animals were similarly plotted on the map.

### Statistical analysis

Qualitative variables were expressed as percentages or proportions. Groups were compared using Fisher's exact test. A test with a *p* value lower than 0.05 was considered statistically significant. Statistical analyses were performed by using Stata 11.0 (StataCorp LP, College Station, TX, USA).

### Sampling authorization, ethics statement, and authorization for study on tribal land

Because rodents are introduced invasive Mammals in New Caledonia, they are legally classified as “dangerous detrimental species” and no particular authorization is required for their capture and study [27]. Protocols for animal experiments were prepared and conducted according to the guidelines of the Animal Care and Use Committees of the Institut Pasteur. The protocol was approved before the start of the experiments by a scientific committee and an animal care committee of the Institute Pasteur in New Caledonia.

To obtain the permission of conducting surveys in tribal lands, custom chiefs were met and the project of the research explained during a public information meeting on leptospirosis. A custom council (directed by the tribe and senior council chiefs) gave the necessary agreement for working on their land. All field studies were conducted with the help of a salaried tribe guide.

## Results

### Meteorological conditions during the survey

**Figure 3:**
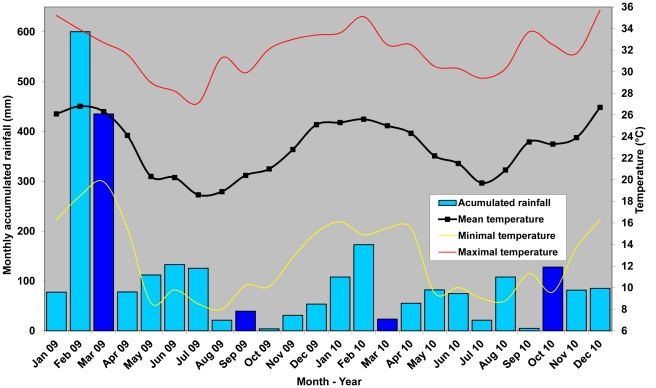
Meteorological data during the two years of the survey (data kindly provided by Météo France). The rainfall bars of our sampling months appear deeper blue.

Fieldwork surveys were carried out in highly contrasted meteorological conditions with major differences in monthly accumulated rainfalls and temperatures (Figure 3): 2009 had a very rainy hot season, while 2010 had a relatively dry hot season. When compared to historical ten-year average rainfall and temperature data, the hot season in 2009 had a large rainfall excess, and stream overflows and floods actually occurred, whereas the 2010 hot season was both cooler and much dryer than a ten-year average. Although the cool season was warmer in 2010 than in 2009, both cool seasons had quite similar patterns, especially regarding (near normal) rainfall intensities.

### Rodent species captured, abundance by species, season and age class

**Figure 4:**
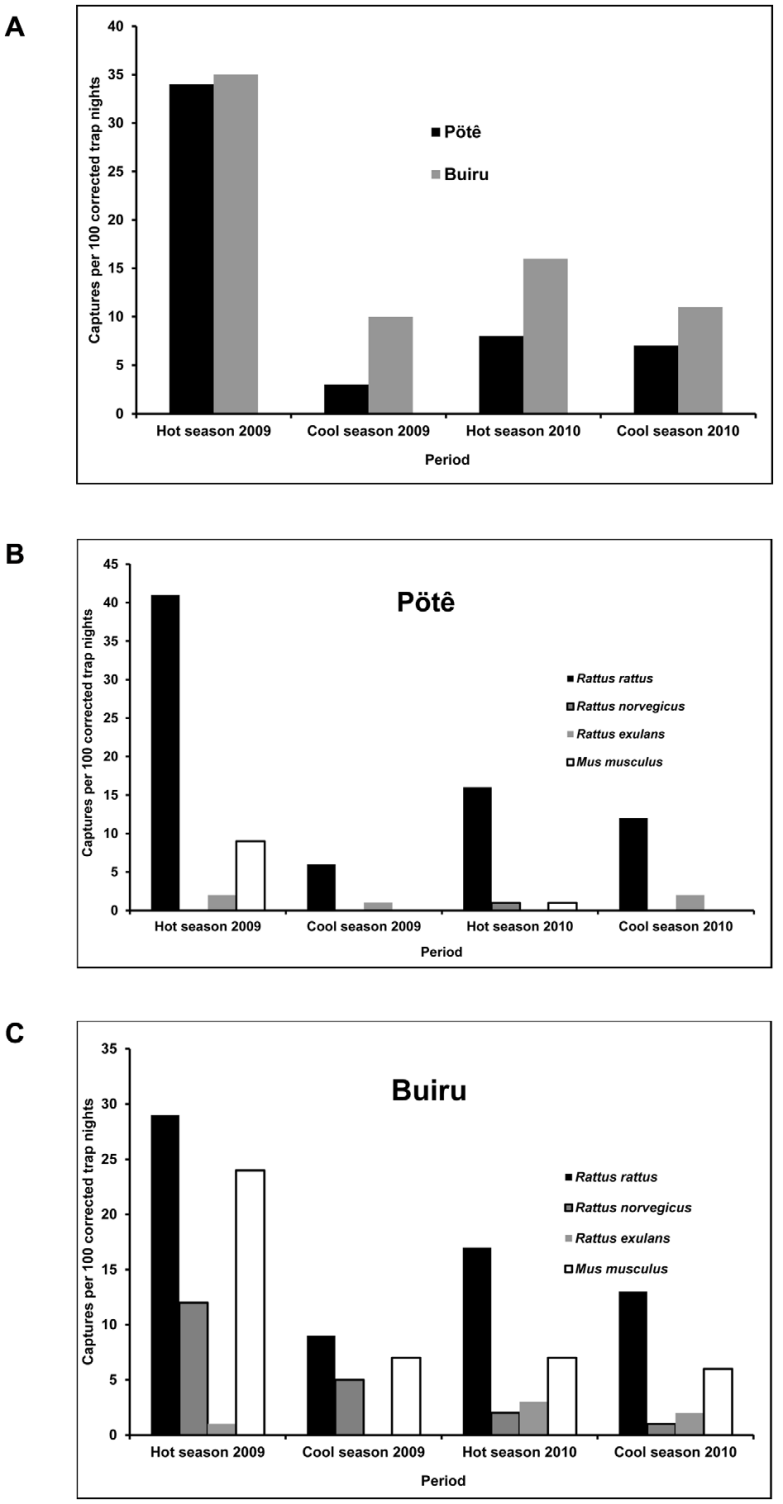
Abundances indexes in each tribe at the different seasons.

All four rodent species known to be present in New Caledonia, namely the three rat species (*R. rattus* the black rat, *R. norvegicus* the brown or Norway rat and *R. exulans* the Polynesian rat) and the domestic mouse (*M. musculus*) were actually captured during our study. A total of 239 rodents were captured, out of which 231 could be identified (species) and 210 could be sampled for *Leptospira* carriage. Similarly due to predation of the captures, the age class could only be ascertained for 213 individuals. The black rat *R. rattus* was the most frequently captured species, accounting for 60.6% of the captures (140 rats), whereas mice *M. musculus* accounted for 25.5% (59 mice), Norway rats *R. norvegicus* for 9.1% (21 individuals), the rarest species being Polynesian rats *R. exulans* (4.8%, 11 rats). The greatest number of captures was achieved during the hot season 2009, with 113 rodents (47.3%). During the 2009 cool season, 2010 hot and cool seasons, the numbers of rodents captured were 28, 56 and 42 respectively. A greater number of rodents were systematically captured in Buiru (a total of 142 captures, 59.4%) than in Pötê (a total of 97 captures, 40.6%). The corresponding values in terms of abundance, an index used to compare rodent densities between different places and seasons and expressed as a number of captured rodents per 100 corrected trap × nights, are shown in Figure 4.

A significantly higher proportion of juveniles was found in all rat species in hot seasons (63.6%) when compared to cool seasons (18.9%) (p<0.001). Contrastingly, in mice, a lower proportion of juveniles was captured in hot seasons (14.3%) compared to cool seasons (46.7%) (p = 0.027).

### *Leptospira* carriage

**Figure 5:**
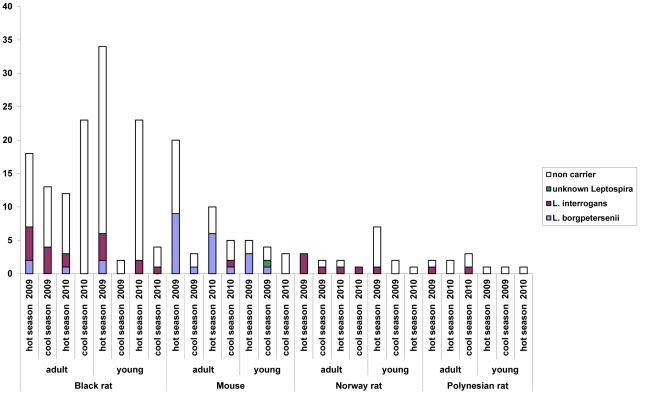
Rodents captured during our study and their *Leptospira* carriage. (The animals for which the stage (adult/juvenile) could not be determined are not represented.).

From the universal 18S amplification, only one kidney DNA extract demonstrated PCR inhibition. This rodent was therefore considered for abundance calculations but excluded from prevalence studies. The detailed results are shown in Figure 5 and Table S2. In total, 56 rodents out of 210 (26.7%) were found as carrying *Leptospira* in their kidneys. This prevalence however considerably varied according to species, age class and seasons. The *Leptospira* prevalence was significantly higher in mice (24/51 = 47.1%) and Norway rats (7/19 = 36.8%) than in black rats (23/129 = 17.8%) or Polynesian rats (2/11 = 18.2%). Considering all species together, adults were more frequently carrying *Leptospira* (40/119 = 33.6%) than juveniles (15/88 = 17%) (p = 0.01), though this difference was not significant in every individual species. As an example, the prevalence was 21.2% in adult black rats, whereas it was 14.3% in juveniles (p = 0.36). Similarly, it was 75% (6 out of 8) in adult Norway rats and only 10% (1/10) in juveniles (p = 0.01).

The culture technique, used only during the first survey (March 2008) allowed the collection of 8 *Leptospira* isolates, from 6 mice (6 isolates), one black rat and one Norway rat. Because of its low yield and frequent failure in *Leptospira* positive kidneys, it was not used in further surveys.

### Temporal trends in *Leptospira* prevalence and effect of age class

The difference in *Leptospira* prevalence between the different surveys (see Figure 5) was not significant. However, trends could be evidenced in all rodent species, regardless of species or age class. The prevalence was higher in hot seasons (30.1%) than in cool seasons (19.4%) this difference however is not significant (p = 0.13). This difference was highest between the hot and rainy season in 2009 (33.7%) than all other seasons grouped together (21.2%) (p = 0.59) and was significant when considering only adult rodents (20/43 = 46.5% vs. 20/76 = 26.3% p = 0.0284). Similarly, the *Leptospira* prevalence was significantly higher during the hot and rainy season in 2009 (31/92 = 33.7%) than during the cool season in 2010 (5/40 = 12.5%) (p = 0.011).

### Isolates identification using MLST

The 8 isolates obtained from mice (6), one Norway rat (1) and one black rat (1) were typed using a MLST scheme described previously [10], [26]. The results pointed to *L. borgpetersenii* putatively belonging to the serogroup Ballum for mice isolates and *L. interrogans* putatively belonging to the serogroup Icterohaemorrhagiae for the isolates from both rat species.

### Strain identification according to the rodent host species

In the 2 Polynesian and 7 Norway rats, the *lfb1* PCR product sequence presumptively identified *L. interrogans* belonging to the Icterohaemorrhagiae serogroup as the infecting *Leptospira* [10]. Identical *lfb1* sequences pointing to the serogroup Icterohaemorrhagiae were obtained from 20 out of 26 (76.9%) black rats, the remaining 6 (23.1%) were infected with a *L. borgpetersenii* with an *lfb1* sequence presumptively pointing to the serogroup Ballum. From 24 *Leptospira*-infected mice, 22 were also infected with a *L. borgpetersenii* presumptively belonging to the serogroup Ballum, one with a *L. interrogans* presumptively identified as serogroup Icterohaemorrhagiae, whereas the last one was infected with an unidentified *Leptospira* sp. Actually, its kidney DNA extract gave a positive PCR amplification using the *lipL32* PCR [24] but failed to be amplified using the other diagnostic PCR targets tested, namely *lfb1* [23], *secY* [28], even if using degenerated primers (see Table S1) or different primers targeting a larger product [29]. Similarly, the TaqMan-based *lipL32* assay [30] gave negative results using this DNA extract. The *lipL32* PCR product was purified and sequenced, yielding a 352 bp sequence (Accession Number JN092329) that did not match any known *Leptospira* strain when compared with sequences available in GenBank using the Blast algorithm. Attempts to specifically amplify the 16S rRNA gene using *Leptospira* specific primers [31] for species identification were conducted on a gradient thermocycler but despite many attempts only allowed the sequencing of 207 bp of this gene. This 16S rDNA sequence (Accession Number JN092330) demonstrated a highest identity of 98% with *L. kirschneri*. The phylogenetic position of this uncultured *Leptospira* as deduced from this short 16S ribosomal sequence is shown in Figure S1.

## Discussion

We were able to capture all four rodent species (the black rat *R. rattus*, the Norway rat *R. norvegicus*, the Polynesian rat *R. exulans* and the mouse *M. musculus*) present in New Caledonia. The Norway rat (*R. norvegicus*) was very rarely captured in Pötê while this species was captured in all four seasons in Buiru. Whatever the site, the black rat (*R. rattus*) was the species most frequently captured and accounted for about 60% of captures. This coexistence of the black rat with Polynesian rats (*R. exulans*) and domestic mice (*M. musculus*) is consistent with previous studies realized in other locations in New Caledonia in uninhabited sclerophylls or rainforests. The sympatric behaviour of these rodent species in New Caledonia is regarded as a peculiarity. The four introduced rodents are usually not been found to coexist in the same habitat notably in New Zealand except possibly on the Chatham Islands [32]. In New Caledonia however, like in Hawaii [33], we found the four species to be sympatric. Our surveys allowed sampling rodents during highly contrasted seasons: the hot season 2009 was especially wet and warm, contrasting with the hot season 2010 which was much drier. The cool seasons 2009 and 2010 were quite similar, except that succeeding to either a wet (2009) or a dry (2010) hot season. Significant differences were noted in rodent abundance, highest abundances being observed during the hot and rainy period in 2009. A marked seasonality of rodent dynamics is well-known and has notably been considered as a major concern when considering rodents as reservoirs of infectious diseases [34], [35] and was modelled for an African rodent in the context of leptospirosis [36]. It is also recognized that seasonal factors must be considered when rodent control programs are to be implemented [37], [38].

The overall prevalence of *Leptospira* spp. in our rodent sample was 26.7%, a finding in accordance with former studies [39], [40]. No correlation was shown between sex and prevalence but age had a major impact on prevalence, adult animals being much more frequently infected (33.6%) than juveniles (17%) (p = 0.01), as already described in other locations [41]–[43]. Mice were more frequently infected than rats (p<0.001), no difference being evidenced between the three rat species (p = 0.16). Interestingly, when considering the ecological habits of the different rodent species, mice and Norway rats that are considered as commensal species (living closer to humans) have a higher prevalence (44.3%) than the more sylvatic black and Polynesian rats (17.9%) (p<0.001). Higher *Leptospira* prevalence in mice and Norway rats compared to black rats was frequently observed in some locations [44], [45], though contrasting results were reported in others [46].

As expected and already largely recognized, mice appeared to maintain *L. borgpetersenii* strains, the DNA sequences pointing to Ballum as the putative serogroup, Norway rats maintaining *L. interrogans* presumptively identified as belonging to the Icterohaemorrhagiae serogroup, both being evidenced in black rats in which Ballum appeared to be less frequent (23%). The simultaneous carriage of these two leptospires in a single (and probably panmictic) black rat population was also already described, e.g. in New Zealand [43] or Argentina [42]. Oppositely, no *L. borgpetersenii* carriage was detected in Norway rats, as was sometimes observed in Hawaii [41] or New Zealand [43]. Only two Polynesian rats were found as carrying leptospires, both from the species *L. interrogans* and presumptively identified as belonging to the serogroup Icterohaemorrhagiae, while leptospires from the serogroup Ballum found in other *R. exulans* populations [41] were not evidenced in our captures.

Unexpectedly, an unknown leptospire was also detected using various PCR techniques. This leptospire was found in the kidney of a domestic mouse. Its sequences clearly point to a species belonging to the pathogenic cluster of *Leptospira* spp. (see Figure S1) but its exact species identification cannot be ascertained. Interestingly, this strain could not be detected using the *lfb1* PCR [23] routinely used for diagnosis in New Caledonia, nor using the TaqMan-based *lipL32* technique [30] or the secY technique [28], all supposed to detect all pathogenic *Leptospira* spp. This surprising finding not only highlights the rich biodiversity of the *Leptospira* phylum but also questions about the existence of other pathogenic *Leptospira* species in New Caledonia that would be undetected using several of the PCR techniques described and currently used for diagnosis.

Water and rodents are known to play pivotal roles in the epidemiology of leptospirosis. Taken together, Icterohaemorrhagiae and Ballum serogroups have been responsible for more than 75% of human leptospirosis cases in New Caledonia [10], again highlighting the major contribution of rodents to human leptospirosis. The increased incidence of human leptospirosis in hot rainy seasons observed in New Caledonia [9], [14] and elsewhere [3] could result from the combined effects of an increased exposure of humans to mud and surface waters and of an increased *Leptospira* contamination of these environments. This latter would also result from both a higher survival probability of leptospires in wet environments during hot rainy periods and higher leptospire abundance due to increased seeding by reservoir populations. We actually evidence a higher rodent abundance and an increased *Leptospira* prevalence in rodent populations during one hot period with heavy rainfall. The results of our study are therefore in agreement with this global scheme, notably suggested as a factor contributing to a leptospirosis epidemics in Guadeloupe, West Indies [47] and with the rural model proposed by Holt and colleagues [36]. Additionally, our study in an area of leptospirosis hyper-endemicity highlights a higher *Leptospira* prevalence in mice and Norway rats, both rodent species which ecology and behavior bring in closer contact to humans compared to the more sylvatic black and Polynesian rats. Taken together, our data strongly suggest that all parameters studied might contribute to the occurrence of human leptospirosis epidemics during hot periods with heavy rainfalls: increased rodent populations with higher *Leptospira* carriage, leading to an increased contamination of an environment more favorable to leptospire survival.

Our data, though in complete agreement with prior knowledge on rodent dynamics elsewhere, only rely on two consecutive years and even more significantly only one season with heavy rain. Because interactions between climate variables, reservoir hosts and the pathogen are especially complex, additional surveys are needed to ascertain the influence of climate on rodents and their *Leptospira* carriage dynamics in the context of New Caledonia. With regard to rodent control measures, our results are also in agreement with previous knowledge and model predictions [36]–[38]. Should the impact of climate and meteorological variability be confirmed, the best rodent management strategy to minimize leptospirosis burden in New Caledonia would probably be the use of rodenticides before the start of a hot rainy period, a situation similar to rodent control for agricultural crops [38], therefore at times of low rodent density and low leptospirosis incidence, also corresponding to periods of low political and public awareness. Nevertheless, because economical modeling tends to demonstrate a similar cost-benefit effect of rodent control measures compared to post-exposure treatments [48], a better control of rodent populations should be increasingly considered as a possible approach for leptospirosis management in endemic areas.

Similarly to a study in Guadeloupe [47], the climatic conditions leading to leptospirosis epidemics in New Caledonia are under strong influence of the El Niño Southern Oscillation [9], [49]. The major advances in the modeling and prediction of this climatic phenomenon probably provides opportunities for predicting leptospirosis epidemics in some regions (e.g. [50]), in turn permitting to implement leptospirosis prevention measures (like river dredging, drainage or rodent control actions) in areas of high leptospirosis endemicity, in a timely manner.

## Supporting Information

Table S1 Primers and probe used in this study and amplicon size.
(PDF)

Table S2 Detailed results of *Leptospira* carriage in kidneys of the rodents captured at different seasons.
(PDF)

Figure S1 **Phylogenetic tree (Neighbour Joining method and Kimura's 2-parameter distances, 500 replicates) inferred from a 207 bp sequence (GenBank Accession Number JN092330) amplified using** ***Leptospira*** **-specific 16S-rDNA primers.** A 352 bp *lipL32* sequence was also deposited in GenBank under Accession Number JN092329.
(PDF)

## References

[1] Baranton G, Old IG (1995). The Spirochaetes: a different way of life.. Bulletin de l'Institut Pasteur.

[2] Adler B, de la Pena Moctezuma A (2009). Leptospira and leptospirosis.. Vet Microbiol.

[3] Levett PN (2001). Leptospirosis.. Clinical Microbiology Reviews.

[4] Desvars A, Cardinale E, Michault A (2010). Animal leptospirosis in small tropical areas.. Epidemiol Infect.

[5] Vinetz JM (2001). Leptospirosis.. Curr Opin Infect Dis.

[6] Reviliod P, Sarasin F, Roux J (1914). Les Mammifères de la Nouvelle-Calédonie et des iles Loyalty.. Nova Caledonia - Forschungen in Neu-Caledonien und auf den Loyalty-Inseln. Recherches scientifiques en Nouvelle-Calédonie et aux iles Loyalty. Nova Caledonia - Forschungen in Neu-Caledonien und auf den Loyalty-Inseln Recherches scientifiques en Nouvelle-Calédonie et aux iles Loyalty.

[7] Gargominy O, Bouchet P, Pascal M, Jaffré T, Tourneur JC (1996). Conséquences des introductions d'espèces animales et végétales sur la biodiversité en Nouvelle-Calédonie. , vol 51. 375–402.. Rev Ecol (Terre Vie).

[8] Perrocheau A, Perolat P (1997). Epidemiology of leptospirosis in New Caledonia (South Pacific): a one-year survey.. Eur J Epidemiol.

[9] Berlioz-Arthaud A, Merien F, Baranton G (2007). Bilan de cinq années de surveillance biologique de la leptospirose humaine en Nouvelle-Calédonie (2001–2005). [Laboratory based human leptospirosis surveillance in New Caledonia (2001–2005).].. Bull Soc Pathol Exot.

[10] Perez J, Goarant C (2010). Rapid *Leptospira* identification by direct sequencing of the diagnostic PCR products in New Caledonia.. BMC Microbiol.

[11] Gaynor K, Katz AR, Park SY, Nakata M, Clark TA (2007). Leptospirosis on Oahu: an outbreak associated with flooding of a university campus.. Am J Trop Med Hyg.

[12] Johnson MA, Smith H, Joeph P, Gilman RH, Bautista CT (2004). Environmental exposure and leptospirosis, Peru.. Emerg Infect Dis.

[13] Chauhan V, Mahesh DM, Panda P, Mokta J, Thakur S (2010). Profile of patients of leptospirosis in sub-Himalayan region of North India.. J Assoc Physicians India.

[14] Goarant C, Laumond-Barny S, Perez J, Vernel-Pauillac F, Chanteau S (2009). Outbreak of leptospirosis in New Caledonia: diagnosis issues and burden of disease.. Tropical Medicine and International Health.

[15] Merien F, Perolat P (1996). Public health importance of human leptospirosis in the South Pacific: a five-year study in New Caledonia.. Am J Trop Med Hyg.

[16] Ko AI, Galvao Reis M, Ribeiro Dourado CM, Johnson WD, Riley LW (1999). Urban epidemic of severe leptospirosis in Brazil. Salvador Leptospirosis Study Group.. Lancet.

[17] Reis RB, Ribeiro GS, Felzemburgh RD, Santana FS, Mohr S (2008). Impact of environment and social gradient on leptospira infection in urban slums.. PLoS Negl Trop Dis.

[18] Soares TS, Latorre Mdo R, Laporta GZ, Buzzar MR (2010). Spatial and seasonal analysis on leptospirosis in the municipality of Sao Paulo, Southeastern Brazil, 1998 to 2006.. Rev Saude Publica.

[19] Bourée P, Benoist L, Perolat P (1999). Etude épidémiologique et clinique de la leptospirose à Bourail (Nouvelle Calédonie).. Bulletin de la Société de Pathologie Exotique.

[20] Cunningham D, Moors PJ (1996). Guide to identification and collection of New Zealand rodents. Department of Conservation ed.

[21] Nelson LJ, Clark FW (1973). Correction for sprung traps in catch/effort calculations of trapping results.. Journal of Mammalogy.

[22] Johnson RC, Rogers P (1964). 5-Fluorouracil as a Selective Agent for Growth of Leptospirae.. J Bacteriol.

[23] Merien F, Portnoi D, Bourhy P, Charavay F, Berlioz-Arthaud A (2005). A rapid and quantitative method for the detection of *Leptospira* species in human leptospirosis.. FEMS Microbiology Letters.

[24] Levett PN, Morey RE, Galloway RL, Turner DE, Steigerwalt AG (2005). Detection of pathogenic leptospires by real-time quantitative PCR.. Journal of Medical Microbiology.

[25] Mahdi LE, Statzell-Tallman A, Fell JW, Brown MV, Donachie SP (2008). Sympodiomycopsis lanaiensis sp. nov., a basidiomycetous yeast (Ustilaginomycotina: Microstromatales) from marine driftwood in Hawai'i.. FEMS Yeast Res.

[26] Thaipadungpanit J, Wuthiekanun V, Chierakul W, Smythe LD, Petkanchanapong W (2007). A Dominant Clone of *Leptospira interrogans* Associated with an Outbreak of Human Leptospirosis in Thailand.. PLoS Negl Trop Dis.

[27] Goarant AC (2010). La délibération n°06-2009 du 18 février 2009 de la province Sud de la Nouvelle-Calédonie relative à la récolte et à l'exploitation des ressources biochimiques et génétiques.. Ethnopharmacologia.

[28] Ahmed A, Engelberts MF, Boer KR, Ahmed N, Hartskeerl RA (2009). Development and validation of a real-time PCR for detection of pathogenic *Leptospira* species in clinical materials.. PLoS One.

[29] Ahmed N, Devi SM, Valverde Mde L, Vijayachari P, Machang'u RS (2006). Multilocus sequence typing method for identification and genotypic classification of pathogenic *Leptospira* species.. Ann Clin Microbiol Antimicrob.

[30] Stoddard RA, Gee JE, Wilkins PP, McCaustland K, Hoffmaster AR (2009). Detection of pathogenic Leptospira spp. through TaqMan polymerase chain reaction targeting the LipL32 gene.. Diagn Microbiol Infect Dis.

[31] Merien F, Amouriaux P, Perolat P, Baranton G, Saint Girons I (1992). Polymerase chain reaction for detection of Leptospira spp. in clinical samples.. Journal of Clinical Microbiology.

[32] King CM, Press OU (2005). The Handbook of New Zealand Mammals..

[33] Sugihara RT (1997). Abundance and diets of rats in two native Hawaiian forests.. Pacific Science.

[34] Mills JN, Childs JE (1998). Ecologic studies of rodent reservoirs: their relevance for human health.. Emerg Infect Dis.

[35] Tagliapietra V, Rosa R, Hauffe HC, Laakkonen J, Voutilainen L (2009). Spatial and temporal dynamics of lymphocytic choriomeningitis virus in wild rodents, northern Italy.. Emerg Infect Dis.

[36] Holt J, Davis S, Leirs H (2006). A model of Leptospirosis infection in an African rodent to determine risk to humans: seasonal fluctuations and the impact of rodent control.. Acta Trop.

[37] Mohan Rao A (2006). Preventive measures for leptospirosis: rodent control.. Indian J Med Microbiol.

[38] Skonhoft A, Leirs H, Andreassen HP, Mulungu LSA, Stenseth NC (2006). The bioeconomics of controlling an African rodent pest species.. Environment and Development Economics.

[39] Suepaul SM, Carrington CV, Campbell M, Borde G, Adesiyun AA (2010). Serovars of Leptospira isolated from dogs and rodents.. Epidemiol Infect.

[40] Bunnell JE, Hice CL, Watts DM, Montrueil V, Tesh RB (2000). Detection of pathogenic Leptospira spp. infections among mammals captured in the Peruvian Amazon basin region.. Am J Trop Med Hyg.

[41] Shimizu MM (1984). Environmental and biological determinants for the prevalence of leptospirosis among wild small mammal hosts, island of Hawaii.. Int J Zoonoses.

[42] Vanasco NB, Sequeira MD, Sequeira G, Tarabla HD (2003). Associations between leptospiral infection and seropositivity in rodents and environmental characteristics in Argentina.. Prev Vet Med.

[43] Hathaway SC, Blackmore DK, Marshall RB (1981). Leptospirosis in free-living species in New Zealand.. J Wildl Dis.

[44] Taylor KD, Turner LH, Everard JD (1991). Leptospires in Rattus spp. on Barbados.. J Trop Med Hyg.

[45] Turk N, Milas Z, Margaletic J, Staresina V, Slavica A (2003). Molecular characterization of *Leptospira* spp. strains isolated from small rodents in Croatia.. Epidemiol Infect.

[46] Rahelinirina S, Léon A, Hartskeerl R, Sertour N, Ahmed A (2010). First Isolation and Direct Evidence for the Existence of Large Small-Mammal Reservoirs of *Leptospira* sp. in Madagascar.. PLoS ONE.

[47] Herrmann-Storck C, Postic D, Lamaury I, Perez JM (2008). Changes in epidemiology of leptospirosis in 2003–2004, a two El Nino Southern Oscillation period, Guadeloupe archipelago, French West Indies.. Epidemiol Infect.

[48] Mendoza RL (2010). Leptospirosis in the tropics: when prevention doesn't easily sell as a ton of cure.. American Journal of Economics and Business Administration.

[49] Hales S, Weinstein P, Souares Y, Woodward A (1999). El Niño and the dynamics of vectorborne disease transmission.. Environ Health Perspect.

[50] Desvars A, Jégo S, Chiroleu F, Bourhy P, Cardinale E (2011). Seasonality of Human Leptospirosis in Reunion Island (Indian Ocean) and Its Association with Meteorological Data.. PLoS ONE.

