# Supplementary material for: Rodent Abundance Dynamics and Leptospirosis Carriage in an Area of Hyper-Endemicity in New Caledonia
Source: PLoS Negl Trop Dis. 2011 Oct 25;5(10):e1361. doi: 10.1371/journal.pntd.0001361 (PMC3201910; doi:10.1371/journal.pntd.0001361)
Supplement: Table S1 — Primers and probe used in this study and amplicon size. (PDF) [file pntd.0001361.s001.pdf]

Supporting Table S1. Primers and probe used in this study and amplicon size

Primers	Reference	Sequence	Amplicon size (bp)
lfb1-F<br>*lfb1*-R	[23]	CATTCATGTTTCGAATCATTTCAAA<br>GGCCCAAGTTCCTTCTAAAAG	331
lipL32-270F<br>*lipL32*-692R	[24]	CGCTGAAATGGGAGTTCGTATGATT<br>CCAACAGATGCAACGAAAGATCCTTT	423
secY-IVFd<br>*secY*-IVRd	modified from [28]	GCGATTCAGTTYAAYCCTGC<br>GAGTTRGARCTCAAATCTAAG	202
18S-F<br>18S-R	[25]	TACCTGGTTGATCCTGCCAGT<br>TTGATCCTTCTGCAGGTTCACCTAC	~1850
lipL32-45F<br>lipL32-286R<br>lipL32-189P	[30]	AAGCATTACCGCTTGTGGTG<br>GAACTCCCATTTCAGCGATT<br>FAM-AAAGCCAGGACAAGCGCCG-TAMRA	242
secY-F<br>secY-R	[29]	ATGCCGATCATTTTTGCTTC<br>CCGTCCCTTAATTTTAGACTTCTTC	549
pfkB-F<br>pfkB-R	[26]	CCGAAGATAAGGGGCATACC<br>CAAGCTAAAACCGTGAGTGATT	559
pntA-F<br>pntA-R		TGCCGATCCTACAACATTA<br>AAGAAGCAAGATCCACAACTAC	637
sucA-F<br>sucA-R		AGAAGAGGCCGGTTATCATCAG<br>CTTCCGGGTCGTCTCCATTTA	559
tpiA-F<br>tpiA-R		AAGCCGTTTTCCTAGCACATTC<br>AGGCGCCTACAAAAAGACCAGA	554
mreA-F<br>mreA-R		GTAAAAGCGGCCAACCTAACAC<br>ACGATCCCAGACGCAAGTAA	601
glmU-F<br>glmU-R		GGAAGGGCACCCGTATGAA<br>TCCCTGAGCGTTTTGATTT	556
fadD-F<br>fadD-R		AGTATGCGTATCTTCCTCCTT<br>TTCCCACTGTAATTTCTCCTAA	576
