# Supplementary material for: Rodent Abundance Dynamics and Leptospirosis Carriage in an Area of Hyper-Endemicity in New Caledonia
Source: PLoS Negl Trop Dis. 2011 Oct 25;5(10):e1361. doi: 10.1371/journal.pntd.0001361 (PMC3201910; doi:10.1371/journal.pntd.0001361)
Supplement: Table S2 — Detailed results of Leptospira carriage in kidneys of the rodents captured at different seasons. (PDF) [file pntd.0001361.s002.pdf]

Supporting Table S2: Detailed results of *Leptospira* carriage in kidneys of the rodents captured at different seasons.

	**Black rat (*R. rattus*)**									
Age class	adult				juvenile				ND	
Season	Hot 2009	Cool 2009	Hot 2010	Cool 2010	Hot 2009	Cool 2009	Hot 2010	Cool 2010	Hot 2009	Cool 2010
carrier	7	4	3		6		2	1		
non carrier	11	9	9	23	28	2	21	3		
Total	18	13	12	23	34	2	23	4		

	**Mouse (*M. musculus*)**									
Age class	adult				juvenile				ND	
Season	Hot 2009	Cool 2009	Hot 2010	Cool 2010	Hot 2009	Cool 2009	Hot 2010	Cool 2010	Hot 2009	Cool 2010
carrier	9	1	6	2	3	2			1	
non carrier	11	2	4	3	2	2		3		
Total	20	3	10	5	5	4		3	1	

	**Norway rat (*R. norvegicus*)**									
Age class	adult				juvenile				ND	
Season	Hot 2009	Cool 2009	Hot 2010	Cool 2010	Hot 2009	Cool 2009	Hot 2010	Cool 2010	Hot 2009	Cool 2010
carrier	3	1	1	1	1					
non carrier		1	1		6	2	1		1	
Total	3	2	2	1	7	2	1		1	

	**Polynesian rat (*R. exulans*)**									
Age class	adult				juvenile				ND	
Season	Hot 2009	Cool 2009	Hot 2010	Cool 2010	Hot 2009	Cool 2009	Hot 2010	Cool 2010	Hot 2009	Cool 2010
carrier	1			1						
non carrier	1	2		2	1	1	1			1
Total	2	2		3	1	1	1			1
