# Supplementary material for: Rodent Abundance Dynamics and Leptospirosis Carriage in an Area of Hyper-Endemicity in New Caledonia
Source: PLoS Negl Trop Dis. 2011 Oct 25;5(10):e1361. doi: 10.1371/journal.pntd.0001361 (PMC3201910; doi:10.1371/journal.pntd.0001361)
Supplement: Figure S1 — Phylogenetic tree (Neighbour Joining method and Kimura's 2-parameter distances, 500 replicates) inferred from a 207 bp sequence (GenBank Accession Number JN092330) amplified using Leptospira -specific 16S-rDNA primers. A 352 bp lipL32 sequence was also deposited in GenBank under Accession Number JN092329. (PDF) [file pntd.0001361.s003.pdf]

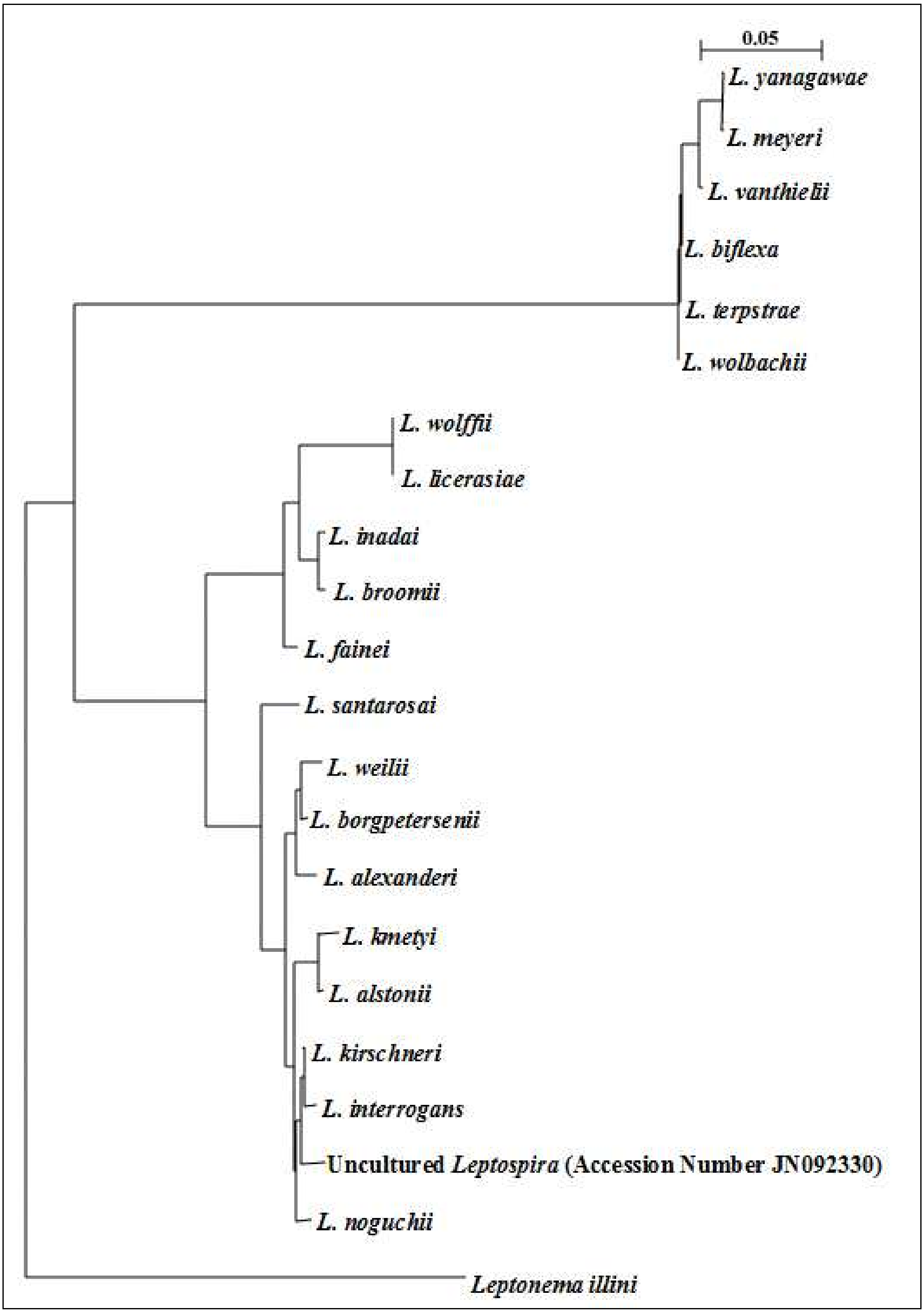

Supporting Figure S1: Phylogenetic tree (Neighbour Joining method and Kimura's 2-parameter distances, 500 replicates) inferred from a 207 bp sequence (GenBank Accession Number JN092330) amplified using *Leptospira*-specific 16S-rDNA primers. A 352 bp *lipL32* sequence was also deposited in GenBank under Accession Number JN092329.
